# Raw Marine Sediments in Concrete: Mechanical Performance, Durability, Environmental Release, and End-of-Life Behavior

**DOI:** 10.3390/ma19183905

**Published:** 2026-09-14

**Authors:** Farjallah Alassaad, Bechara Haddad, Houssam Affan, Ilyas Ennahal, Walid Maherzi, Amro Yaghi, Anas Issa

**Affiliations:** 1 College of Engineering and Technology, American University of the Middle East, Egaila 54200, Kuwait; amro.yaghi@aum.edu.kw (A.Y.); anas.issa@aum.edu.kw (A.I.); 2 Unité de Recherche “Builders Lab”, Builders Ecole d’Ingénieurs, ComUE NU, Campus Caen, 14610 Epron, France; bechara.haddad@builders-ingenieurs.fr; 3 Groupe FEHR, Laboratoire Béton, 62 Route de Strasbourg, 67242 Bischwiller, France; houssam.affan96@gmail.com; 4 Institut Mines-Télécom, 1015 Rue Charles Bourseul, University of Lille, 59500 Douai, France; ennahal-i@live.fr (I.E.); walid.maherzi@imt-nord-europe.fr (W.M.)

**Keywords:** raw marine sediment, sand replacement, concrete durability, environmental leaching, end-of-life behavior, circular economy

## Abstract

**Highlights:**

- Raw marine sediments replaced up to 30% of natural sand in concrete.
- Sediment addition increased porosity, shrinkage, and carbonation depth.
- Preliminary single-slab runoff results remained below the adopted screening limits.
- Crushing remobilized fluorides and nickel, revealing end-of-life risks.

**Abstract:**

The valorization of raw dredged sediments in concrete requires simultaneous verification of engineering performance and environmental safety during both service life and end-of-life conditions. This work focuses on raw marine sediment collected from a port area as a partial substitute for natural sand, through a global assessment of physical properties, mechanical performance, durability, simulated-rainfall release, and leaching after crushing. Four concretes with 0, 10, 20, and 30% of sediment, with the same contents of cement and effective water, and targeting the same SF2 consistency class, were manufactured. Increasing the replacement rate increased porosity, water absorption, shrinkage, and carbonation depth, while lowering the compressive strength. At 28 days, the compressive strength dropped from 43.91 MPa for the reference to 38.16 MPa at the 20% replacement and 24.63 MPa at the 30% replacement. The apparent chloride migration coefficient decreased by about 31 and 77% for the two replacement rates, respectively; given the initial chloride content of the sediment, this trend is interpreted comparatively and possible mechanisms are discussed cautiously. In the simulated-rainfall test, releases of anions, trace metals, and organic indicators measured from the intact concrete remained below the adopted screening thresholds; however, because only one slab per formulation was tested, these environmental observations are preliminary. After crushing, some sediment-containing mixtures exceeded inert-waste thresholds for chlorides, fluorides, or nickel, showing that stabilization was incomplete under end-of-life conditions. Among the sediment-containing mixtures tested, the 20% replacement provided the best overall balance between natural-resource substitution, technical performance, and the preliminary environmental observations.

## 1. Introduction

The increased depletion of mineral resources, large volumes of extracted and/or dredged materials, and stringent environmental legislation have prompted a reconsideration of the potential of sediments as an alternative source of construction materials. In a circular economy approach, valorizing these sediments provides two main advantages: first, it reduces the demand for natural resources, specifically riverbed deposits, and second, it reduces the costs and logistics involved in their handling and management. This issue is especially important for dredged sediments since they are produced in large amounts in rivers, ports, and lakes [1,2].

Despite these benefits, sediments should not be viewed as a classical mineral resource. Valorization of sediment wastes is difficult due to the wide variety in their chemical composition, as well as the possible presence of a large number of fine-fraction particles, high moisture content, organic and salt material, and mineral or metal pollutants. It has been noted that this makes such waste materials potentially harmful, as the smallest fractions tend to affect the chemical composition of cement, water requirements, and mechanical strength characteristics of the materials produced [3,4,5,6,7,8,9]. Moreover, from an ecological point of view, the use of sediment materials entails assessing the risk of their potential release into the environment.

In this regard, the use of sediments in cementitious matrices has already become an extensively studied topic. Namely, the research has investigated not only the addition of treated or calcined sediments but also their partial replacement for fine aggregates in mortars and concretes. As demonstrated by multiple studies, depending on the type of sediment, its preparation state, and the rate of its inclusion, performance levels sufficient for a particular application can be reached [1,10,11,12,13]. For instance, according to the results reported by Ozer-Erdogan et al. [14], it became clear that it is possible to consider dredged marine materials as an alternative to fine aggregates in ready-mix concrete; however, it was found that pretreatment was necessary. In turn, Beddaa et al. [15] showed that some fractions of sediments could be used to create new concrete constructions without much harm to their properties, while other, mainly fine fractions containing much silt and soluble organic material, proved harmful to concrete. Meanwhile, Amar et al. [10] have found that reasonable amounts of treated sediments may provide positive changes in terms of some sustainability indicators in cementitious matrices.

In addition to performance during use, the literature further shows that the cementitious matrix may be capable of providing a solidification/stabilization effect on contaminants existing in the sediments. According to the study by Zhang et al. [16] on contaminated marine sediments stabilized with cement and slag, the immobilization of various elements may become critical in cementitious matrices, especially at pH values suitable for such materials. This characteristic gives sediment incorporation an additional edge over mere granular replacement, as it helps reduce the mobility of various pollutants. Caution should nevertheless be taken regarding the limits of such stabilization effects. On the one hand, they are subject to the types of contaminants, formulation, curing, and exposure conditions; on the other hand, they still entail some environmental risks, especially when the material is exposed to multiple stress cycles, degradation processes, or changing states at the end of its life span. In other words, its use in concrete can constitute a form of partial environmental stabilization, but not a complete neutralization of the problems associated with raw sediments [1,16].

Recent research has already documented several essential dimensions of the issue: the potential of sediment valorization in cementitious materials, the influence of their composition on performance properties, sustainability challenges, and release behavior in stabilization/solidification approaches or environmental assessments [1,2,6,7,8,9]. However, fewer studies examine, within the same experimental protocol and using untreated or minimally processed sediments, the combined relationship between granular substitution, mechanical performance, durability, release from intact concrete under simulated rainfall, and end-of-life leaching after crushing. This gap is important because the acceptability of a recovery pathway cannot be assessed solely based on the behavior of concrete in its unaltered state. It requires a multi-criteria analysis that considers technical performance, environmental stability in service, and the potential evolution of risk when the material reaches its demolition or recycling phase. In this context, the scientific challenge does not lie in proving the general possibility of incorporating sediments into cement-based matrices, since the issue is already well documented in the literature, but rather in identifying, for a specific raw-sediment case study, the highest tested incorporation level that maintains an acceptable balance among the relevant variables.

The current study fits within this paradigm by providing an extensive experimental evaluation of concrete mixes using crude sediments as partial replacements for fine aggregate. Building on the existing literature, this study jointly analyzes mechanical properties, durability, release under simulated rainfall from intact concrete, and post-crushing environmental behavior within the same experimental program.

The objective is, therefore, not to claim a conceptual break from previous work, but to provide applied, multi-criteria insights into the conditions for utilizing raw sediments in concrete. Such an approach aims to better identify a practically acceptable range of substitutions. This range is defined here as one in which incorporation simultaneously enables a reduction in the use of natural resources, an acceptable level of technical performance, and a limited risk of release under the investigated conditions, including the post-crushing stage.

## 2. Materials

The material-property data reported in Table 1, Table 2, Table 3 and Table 4 were obtained from supplier/manufacturer data sheets, whereas the sediment characterization and environmental data in Table 5, Table 6 and Table 7 were obtained from an external laboratory.

### 2.1. Cement

The binder used in this study is Portland cement CEM II/A-LL 52.5 R, chosen for its high early-age strength and rapid hardening, properties particularly suited to concrete formulations intended for structural or prefabrication applications. The cement characteristics can be found in Table 1.

### 2.2. Limestone Filler

Limestone filler was added for enhancing the particle packing effect and optimizing technical and economical performance of the mixture. The material is composed mainly of CaCO_3_ (98.2%) and chloride, sulfate and total silica contents are extremely low (0.003%, 0.01%, and 0.02%, respectively). Therefore, the filler is expected to behave mainly through physical filling and nucleation effects instead of pozzolanic activity. The high fineness of this filler with 98% of particles below 0.125 mm and 90% below 0.063 mm may be responsible for a denser paste and improved early-age hydration [17]. The density of the limestone filler was equal to 2700 kg/m^3^ (summarized in Table 2).

### 2.3. Fine Aggregate

The reference fine aggregate was a washed 0/4 natural sand, selected as the control material prior to incorporating sediment-derived aggregates. This sand was chosen due to its consistent grading and cleanliness, ensuring reliable baseline performance for comparison. Its low water absorption indicates limited porosity, while the relatively high sand equivalent value reflects a low content of clay-like fines. The fineness modulus suggests a well-graded material suitable for concrete applications. Its main physical properties are presented in Table 3.

### 2.4. Coarse Aggregates

The coarse aggregate consisted of locally sourced, washed, semi-crushed gravel with a maximum particle size of 12.5 mm. This type of aggregate was selected for its good mechanical performance and suitability for structural concrete. The relatively low water absorption indicates moderate porosity, while the Los Angeles coefficient reflects good resistance to fragmentation under mechanical stress. The freeze–thaw resistance value suggests limited degradation under cyclic environmental conditions. Its main physical properties are presented in Table 4.

### 2.5. Sediment Characterization

The sediment studied is a natural marine sediment extracted from a port in Normandy on the English Channel coast. It has been used as a partial replacement for sand in concrete. Its characterization first highlights very high content of fines. Indeed, the percentage of particles smaller than 0.063 mm is 83.3%, and the content of fines is 84.9% (Table 5). The characterization of the sediment also confirms its fine nature, with 46.0% of particles smaller than 22 µm and 19.3% smaller than 8 µm (Table 5). These results are in agreement with the literature, which describes dredged sediments as generally fine, heterogeneous material with higher contents of silt and clay as compared to the natural sands that are added to concrete [1]. This remark is important for the topic discussed in this article. It can change the granular structure, increase water consumption, and affect mechanical properties if the addition rate increases. Beddaa et al. (2020) have proved that the finest fractions are the worst for cement hydration and concrete performance [15].

The chemical analysis of the sediment indicates that the main constituent is silica, with SiO_2_ as the primary oxide, accounting for 56.59%. In contrast, the contents of Al_2_O_3_, Fe_2_O_3_, and CaO oxides remain relatively low, at 2.33%, 2.65%, and 2.73%, respectively (see Table 5 and Table 6). The sediment also consists of 1.508% water-soluble chlorides, 0.34% soluble sulfates, and 8.84% humic compounds. Loss on ignition at 550 °C constitutes 10.64% (see Table 5 and Table 6). All these parameters are typical of marine raw sediments, which involve issues with salt, fine material, and organic content [1]. They are especially important for industrial applications. Chlorides and sulfates pose sustainability risks, whereas organics and fines may slow hydration and hinder setting, as evidenced by the influence on the setting time test, during which an additional 140 min was observed (Table 5).

Physical and geotechnical properties also pose sustainability risks. The water content of the sediment is 22.1%, while water absorption stands at 4.3%. The liquid limit is 62, and the plasticity index is 20 (Table 5). The sediment is thus classified as a rather fine, plastically active, and relatively absorbent material compared to typical sand. Such classification allows distinguishing this sediment significantly from the fine aggregate usually used in concrete. Nevertheless, its water content is lower than the expansive values reported in the literature for raw sediment samples [1]. Finally, the environmental impact evaluation indicates that this sediment cannot be classified as non-hazardous or inert material and shows exceedances in some leaching parameters, such as chlorides, the soluble fraction, fluorides, sulfates, and molybdenum (Table 7). This conclusion is logical, considering the marine origin of the sediment discussed here, and fully supports the concept proposed in the article. However, it is not enough to substitute part of the natural sand with the new material.

### 2.6. Concrete Mix Design

The sediment was used without washing, thermal treatment, chemical treatment, or stabilization. Before incorporation, it was air-dried during storage. During mixing, the gravel and sediment were first mixed for 1 min so that aggregate-to-aggregate action disaggregated the sediment before the remaining constituents were added. In this manuscript, the term “raw sediment” refers to sediment used without washing, thermal treatment, chemical treatment, or stabilization; natural air-drying during storage and in-mixer disaggregation are regarded as handling steps rather than separate sediment treatment. Four mix designs were developed: a reference concrete without sediment (Ref) and three concretes containing 10%, 20%, and 30% sediment (Sed10, Sed20, and Sed30), respectively, as a sand substitute. The mix compositions are presented in Table 8.

The cement, effective water, and coarse aggregate contents were held constant in order to keep the same w/c ratio and to compare sediment-containing formulations. The total sand–sediment content was also held constant, and the proportion of sediment substitution to the reference sand was increased. The limestone filler and admixture dosages were adjusted in order to compensate for changes in fines content and rheology and to target the same initial consistency class SF2. Such adjustments are commonly required when incorporating sediments because of their fineness, water absorption, and possible organic matter content [1,15,18]. Because sediment content, limestone filler content, and admixture dosage co-vary, the observed differences represent the response of the adjusted formulations and cannot be attributed exclusively to sediment as a single independent variable. The mixtures were comparative laboratory formulations and were not designed as compliance mixtures for a specific exposure class or structural application. The sediment moisture content and water absorption were accounted for when dosing the mixing water: the total water added was calculated from the target effective water by subtracting the water contributed by the measured sediment moisture content and adding the water required to compensate for sediment absorption, while the effective water was maintained at 150 kg/m^3^. The mixing sequence was gravel and sediment for 1 min, followed by sand, then the cementitious materials for 1 min, and finally water and admixture for 1 min. SF2 was used as the fresh-state acceptance criterion; individual slump-flow values were not retained because the test served as a conformity criterion rather than a response variable. Although the mixtures were comparative rather than compliance designs for a specific exposure class, they were conceived for structural/precast concrete applications, and SF2 was selected from the outset to reflect industrial self-compacting concrete practice.

The sediment contains 1.508% water-soluble chloride (Table 6). Based only on the sediment contribution and the mix proportions in Table 8, this corresponds to approximately 1.34, 2.67, and 4.00 kg Cl^−^/m^3^ for Sed10, Sed20, and Sed30, respectively, or about 0.41%, 0.81%, and 1.21% of the cement mass. These estimates use the water-soluble chloride measured in the sediment and do not include chloride contributions from the other constituents. The comparison with EN 206 is therefore approximate, because total concrete chloride must account for chloride contributions from all constituents. EN 206 expresses concrete chloride content as chloride ions by mass of cement and includes reinforced-concrete classes up to Cl 0.40 [19]; on this approximate basis, Sed10 is approximately at this level from the sediment contribution alone, while Sed20 and Sed30 exceed it. The present formulations should consequently not be considered directly suitable for conventional reinforced concrete without additional chloride control, treatment, and verification of the total concrete chloride content. The effective water content was kept at 150 kg/m^3^ for all formulations; no increase in effective water was used to compensate for sediment absorption, while admixture dosage was adjusted to target SF2 consistency.

## 3. Methods

Unless otherwise stated, specimens were demoulded after 24 h and stored in a humid chamber at 20 °C and >90% relative humidity until the scheduled test age. Shrinkage specimens were the exception: shrinkage monitoring began immediately after demoulding under the environmental conditions specified in Section 3.2. Three replicate specimens were tested per formulation for porosity/absorption, shrinkage, chloride migration, carbonation, internal sulfate swelling, and crushed-specimen leaching; the simulated-rainfall test used one slab per formulation. Error bars reported in the figures represent standard deviation.

### 3.1. Water-Accessible Porosity and Water Absorption by Immersion

Water-accessible porosity was determined in accordance with the standard NF P 18-459 [20]. The test specimens were first subjected to vacuum saturation in a water-filled desiccator for a minimum period of 24 h. Following saturation, the samples were subjected to hydrostatic weighing, followed by air weighing. The samples were then subjected to oven-drying at a temperature of 105 ± 5 °C until a constant mass was achieved. This was defined as two successive weightings, spaced 24 h apart, that differed by no more than 0.05%. Water-accessible porosity was obtained using formula (1).

(1) $$Porosity=\frac{M_{a}-M_{d}}{M_{a}-M_{w}}$$

$M_{d}$: Dry mass (g);

$M_{a}$: Wet mass in air (g);

$M_{w}$: Wet mass in water (g).

Water absorption was measured by immersion according to a specific experimental protocol, quantifying the change in the specimen’s mass between its dry and wet states. The absorption parameter is calculated using Equation (2).

(2) $$Water\;absorption=\frac{M_{s}-M_{d}}{M_{d}}\times 100$$

$M_{d}$: Dry mass (g);

$M_{s}$: Saturated mass (g).

These two parameters were studied because they provide complementary information on the porous structure and the hydric behavior of concretes incorporating sediments.

### 3.2. Total Shrinkage

The NF P 18-427 standard [21] provides a protocol for the measurement of total shrinkage. This protocol involves the measurement of prismatic specimens with dimensions of 7 cm × 7 cm × 28 cm, conducted at a relative humidity of 65%. The shrinkage evolution was monitored over time for the different formulations studied. This test was chosen to compare the effect of incorporating sediment on the delayed deformations of concrete.

### 3.3. Compressive Strength

Compressive strength was determined in accordance with standard NF EN 12390-3 [22]. Tests were carried out on cylindrical concrete specimens (Ø11 cm × 22 cm), with three samples per mix design and per time interval, to ensure a satisfactory representation of experimental variability. Before loading, the specimens underwent preliminary surface preparation to ensure flat contact surfaces and to limit support-related biases during the test.

### 3.4. Chloride Ion Migration

Chloride ion penetration resistance was evaluated using an accelerated migration test performed in accordance with standard XP P 18-462 [23]. Tests were conducted on different formulations at the intervals specified in the experimental program.

**Figure 1:**
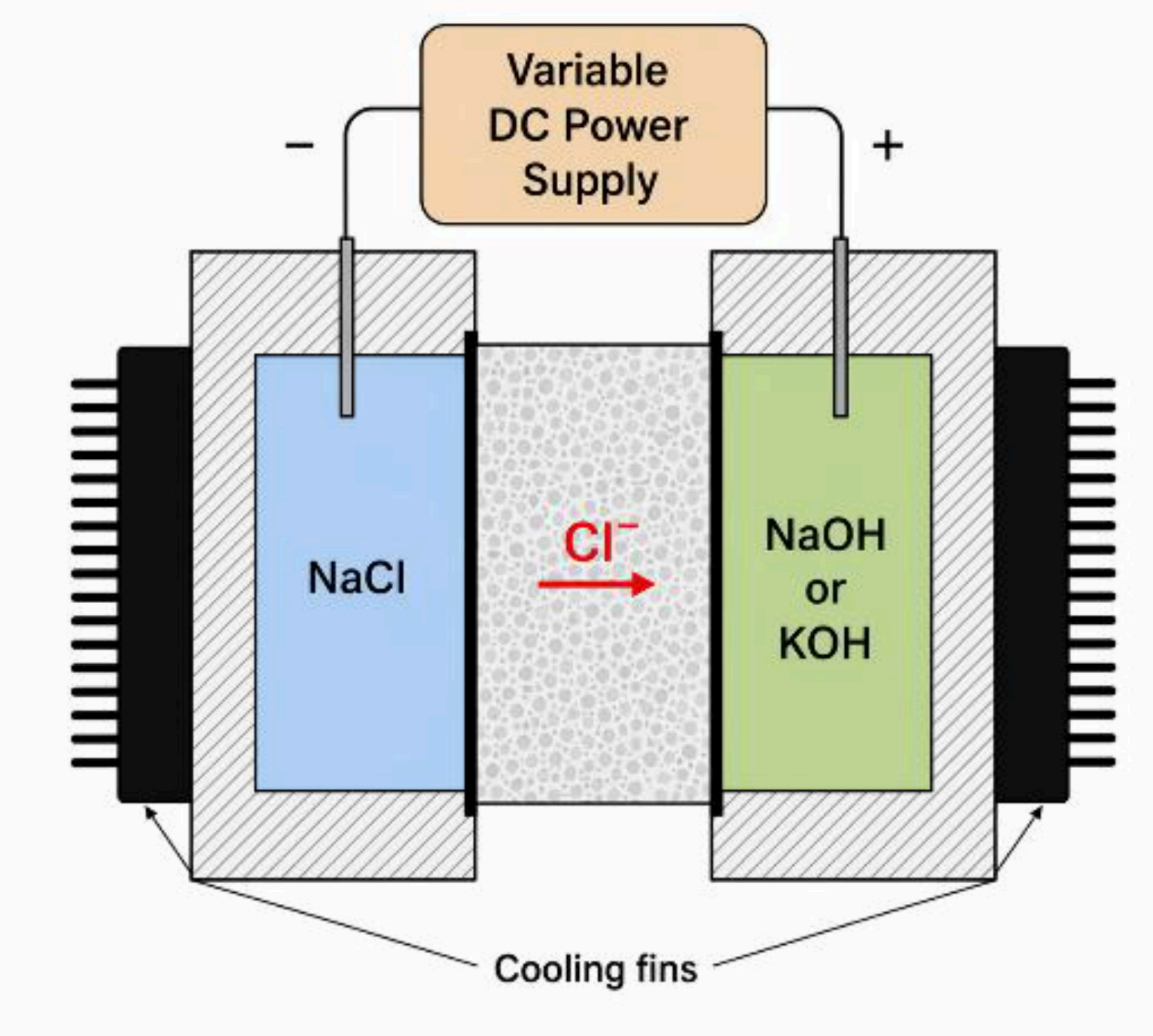
Illustration of chloride ion migration test principle.

This result was achieved through the application of a potential difference of 30 volts between the ends of the samples, as shown in Figure 1. The value obtained was analyzed using the apparent chloride diffusion coefficient (m^2^/s), consistent with the terminology of XP P 18-462 [23]. Because the sediment already contains water-soluble chlorides and the current scope of XP P 18-462 cautions against chloride-contaminated specimens, the values are interpreted here as comparative indicators between the tested formulations rather than as direct compliance values. This test was chosen to assess the effect of sediment-containing formulations on chloride-ion migration.

### 3.5. Accelerated Carbonation

The test specimens were placed in a sealed carbonation chamber in accordance with standard XP P 18-458 [24] under a controlled atmosphere of 3% CO_2_, at 20 °C, and 65% relative humidity. The carbonation depth was measured at various intervals of the experimental program. After removal from the chamber, the specimens were split open, and the carbonated zone was revealed by spraying with a 1% phenolphthalein alcoholic solution. The carbonation depth was then determined in millimeters. This test was chosen to evaluate the effect of sediment incorporation on the susceptibility of concrete to carbonation.

### 3.6. Internal Sulfate Swelling

In the absence of a specific French standard for evaluating internal sulfate attack, the test was conducted in accordance with the recommendations of LPC Test Method No. 66 (2007) regarding the reactivity of concrete to this condition. The protocol is based on successive drying/wetting conditioning, including drying in a climate chamber at (38 ± 2) °C and relative humidity below 30%, followed by immersion. Such conditioning aims to accelerate the formation of expansive products, especially ettringite, and to compare the susceptibility of the mixtures to internal sulfate reactions. Expansion was reported as absolute length change in millimeters and as percentage strain, calculated as ε (%) = (ΔL/L_0_) × 100, with L_0_ = 280 mm, at 28, 60, and 90 days. The procedure is used here as a comparative screening method and should not be interpreted as directly equivalent to a standardized external sulfate-attack test. Prismatic specimens (7 cm × 7 cm × 28 cm; n = 3 per formulation), equipped with fixed measurement studs, were subjected to two conditioning cycles, each consisting of 7 days of drying at 38 ± 2 °C and <30% relative humidity followed by 7 days of immersion in tap water at 20 ± 2 °C. After these 28 days of cyclic conditioning, the specimens were maintained under permanent immersion and expansion was monitored for 6 months. Longitudinal deformation was measured with an extensometer/comparator using the fixed measurement studs. The initial reference length L_0_ used for strain normalization was the full specimen length, 280 mm. The measurement taken at the end of the second immersion cycle was used as the zero/reference reading for the subsequent length-change measurements.

### 3.7. Leaching of Crushed Specimens

In line with EN 12457-2, the leaching tests were conducted on the crushed concrete samples [25]. The crushed material was contacted with water under the conditions defined by the standard, and the resulting leachate was then analyzed to determine the concentrations of anions (including chlorides, sulfates, nitrates, and fluorides) and trace metals. The results were expressed as mass concentrations in the leachate. The test was conducted at 28 days and again after the environmental cycle described in the next section. This test was chosen to assess the environmental behavior of concrete at the end of its life after crushing. As a single-stage compliance leaching test, EN 12457-2 does not characterize pH-dependent release; the present results are therefore limited to the standard test condition.

Accordingly, pH-dependent leaching curves and long-term cumulative release were not derived from the present dataset. These measurements would require a dedicated experimental program and are identified as future work rather than inferred from the single-stage compliance test.

### 3.8. Environmental Monitoring Under Simulated Rain

**Figure 2:**
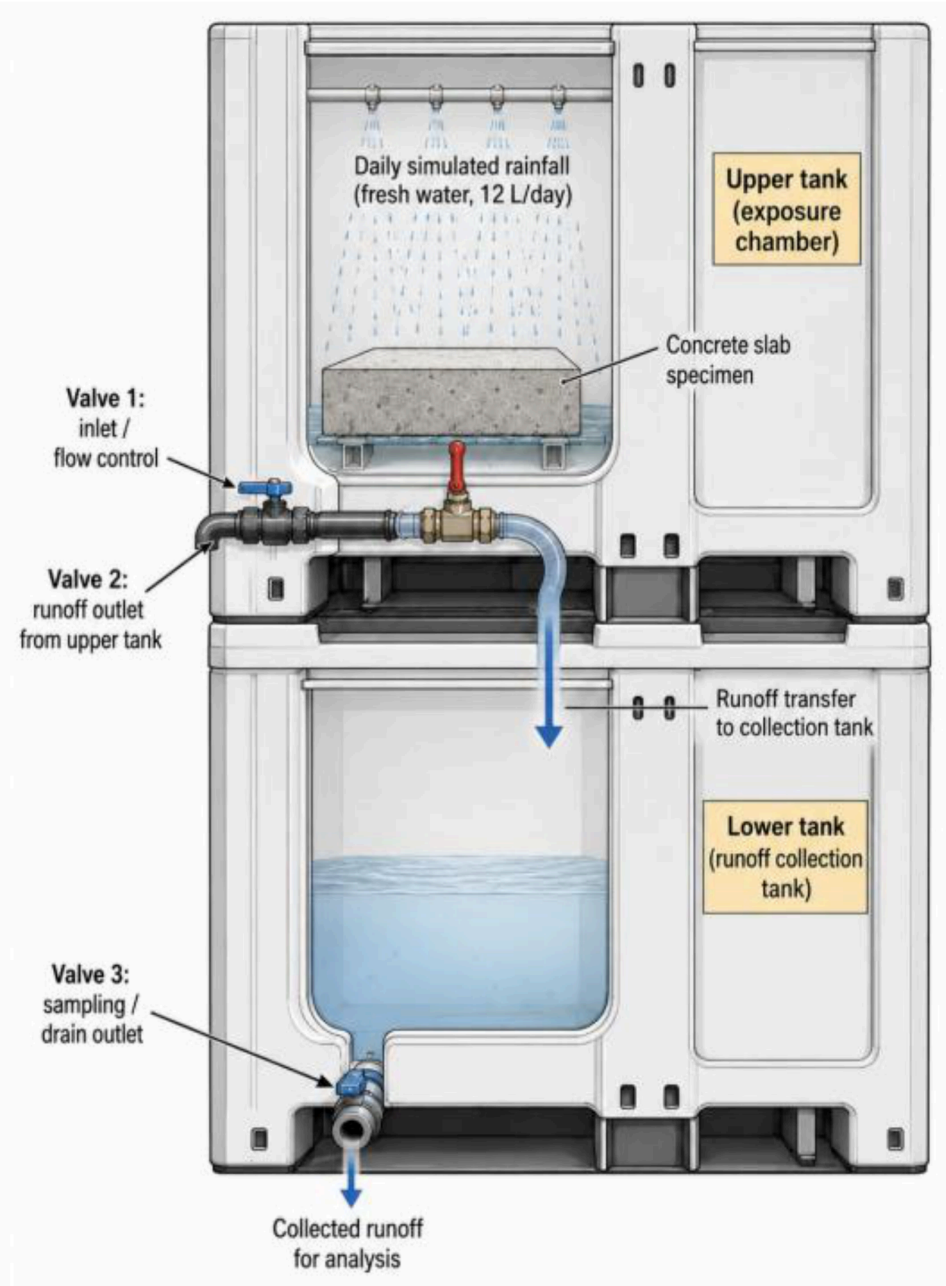
Schematic of the single-pass simulated rainfall setup used for environmental assessment of concrete slabs (redrawn by AI (OpenAI ChatGPT-4o)).

The environmental behavior of the concrete was assessed using a simulated rainfall monitoring system based on exposing experimental concrete slabs to accelerated artificial precipitation, as illustrated in Figure 2. For each slab, 12 L of fresh potable tap water from the Villeneuve-d’Ascq (France) water network was applied daily over an area of 0.25 m^2^. The runoff was collected for monitoring and sampling and was not recirculated. Fresh water was used for each daily application, and the test comprised 139 consecutive rainfall-application days, consistent with the final sampling time reported in consistent with the final sampling time reported in Section 4.8.

This hydraulic protocol corresponds to 48 L/m^2^ per exposure day and to a cumulative applied water volume of approximately 6672 L/m^2^ over 139 days as mentioned in Table 9. The reference annual rainfall was set at 935 L/m^2^/year, corresponding to the 1991–2020 mean annual precipitation aggregated over France, according to Météo-France [26]. This is equivalent to approximately 7 years of rainfall volume. This equivalence is based on cumulative water volume only and does not reproduce natural rainfall intermittency, chemistry, weathering, or the full service life of a concrete structure. Water samples were taken from the runoff collection container after 1, 3, 6, 13, 27, 41, 55, 83, 111, and 139 days and were packaged in bottles designed specifically for environmental analysis.

The innovative concrete was compared to a control concrete subjected to the same protocol. The concentrations measured in the runoff water were interpreted in accordance with the environmental quality standards set by the decree of 11 January 2007, concerning the quality limits of surface freshwater intended for drinking water production. The parameters analyzed included anions (chlorides, sulfates, nitrates, fluorides), trace metals, total organic carbon (TOC), PCBs, PAHs, BTEX, COD, BOD_5_, suspended solids, total hydrocarbons, phenol index, and total nitrogen. For parameters not covered by the decree of 11 January 2007, namely BTEX, TOC, PCBs, molybdenum, nickel, and antimony, interpretation was based on the thresholds defined by the decree of 12 December 2014, concerning the acceptance of inert waste. These values are used as screening benchmarks rather than direct regulatory compliance criteria for concrete runoff: the surface-water decree provides a conservative water-quality reference, while the inert-waste decree provides thresholds for parameters not covered by the former. When measured concentrations consistently remained below the established thresholds, monitoring was discontinued for some parameters; however, analyses of anions and trace metals were continued through the end of the program.

## 4. Results

### 4.1. Water-Accessible Porosity and Water Absorption by Immersion

The principal physical properties of the investigated concretes, wherein some sand is replaced by untreated sediments, are given in Table 10. As can be observed from the presented indicators, a certain regularity is evident with increasing sediment quantity in the mixture. Namely, the value of water-accessible porosity increases gradually until reaching a 20% substitution rate of sand; afterward, it sharply rises to the value of 14.27%. Alongside this increase, the opposite occurs for apparent density: its value decreases from 2443 kg/m^3^ to 2376 kg/m^3^. The same tendency is evident from changes in the value of water absorption during immersion.

According to the findings, when the amount of sediment replacement exceeds 20%, the concrete’s accessible pore structure changes markedly. This trend may be related to the high fines content and water-absorbing capacity, as well as the presence of organic materials and salts in the sediments, which were noted earlier during the characterization process. These characteristics may affect granular packing and local water demand and contribute to increased porosity upon curing. The decrease in density is consistent with the increase in porosity. Therefore, both the increase in open porosity and the absorption during immersion indicate that the matrix becomes more permeable.

These tendencies are predominantly aligned with existing literature. Beddaa et al. showed that fine fractions of dredged sediments, particularly when they retain silt and organic matter, degrade the behavior of concrete more than sandier fractions [27]. Similarly, Berredjem et al. observed an increase in water-accessible porosity and greater vulnerability to water transfer in concretes incorporating fine recycled aggregates [11]. Conversely, more favorable results have been reported when sediments are treated and used as cementitious constituents rather than as sand substitutes: Safhi et al. obtained porosity properties close to the control with up to 20% treated marine sediments after formulation optimization [28]. This difference highlights that the results obtained here are strongly linked to the choice to use raw sediment as a granular substitute.

### 4.2. Total Shrinkage

**Figure 3:**
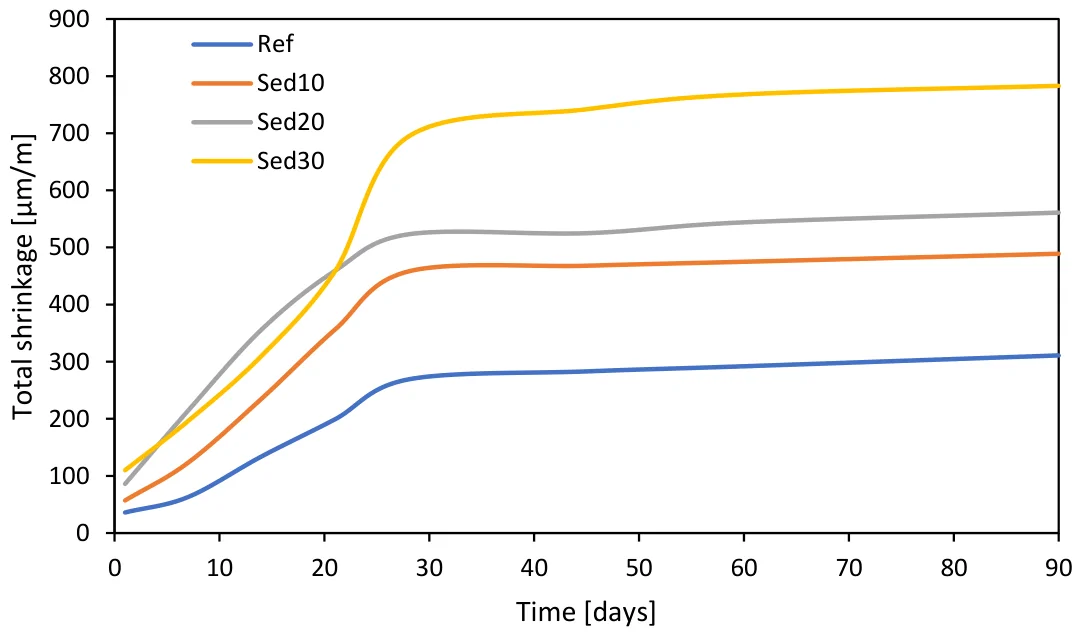
Total shrinkage for the different formulations.

Figure 3 shows the evolution of total shrinkage across the different formulations up to 90 days. Overall, shrinkage increases over time for all concretes and with the sediment incorporation rate. The reference concrete reaches a shrinkage of 311 µm/m, while the Sed10, Sed20, and Sed30 formulations reach 489 µm/m, 561 µm/m, and 783 µm/m, respectively, at 90 days. At 90 days, the shrinkage is more than 2.5 times greater than that of the control. However, most of the concrete shrinkage occurs in the first 28 days.

From these observations, sensitivity to shrinkage increases with increasing sediment substitution, with some degradation occurring up to 20%, while substantial degradation is observed at 30%. This trend may be associated with several factors. Firstly, the relatively fine sediment composition and the high water absorption properties alter moisture transfer within the cement mixture and increase drying deformation. Secondly, organic material and fine-grained components in sediments may interrupt the hydration process, making the matrix more porous and deformable. Special attention should be paid to the initial stages, during which Sed20 exhibited slightly higher shrinkage than Sed30 at both 7 and 14 days. However, in terms of medium-term effects, Sed30 showed the greatest shrinkage.

This evidence is consistent with previous research in this field. As shown by Beddaa et al., using untreated fine sediment rather than sand may lead to an increase in shrinkage up to 200%, but if used in smaller amounts, fine sediments can have significantly fewer negative impacts [18]. Similarly, Beddaa et al. have reported that a 30% substitution with sandy sediment fractions, after removing some of the silt and organic matter, increased shrinkage by only about 15% [27]. The difference in the results obtained here suggests that the coarseness of the sediment studied, its high fines content, and its water retention capacity play a major role. More broadly, the literature on alternative fine aggregates also shows that increased porosity and water absorption are generally accompanied by increased shrinkage [29].

### 4.3. Compressive Strength

**Figure 4:**
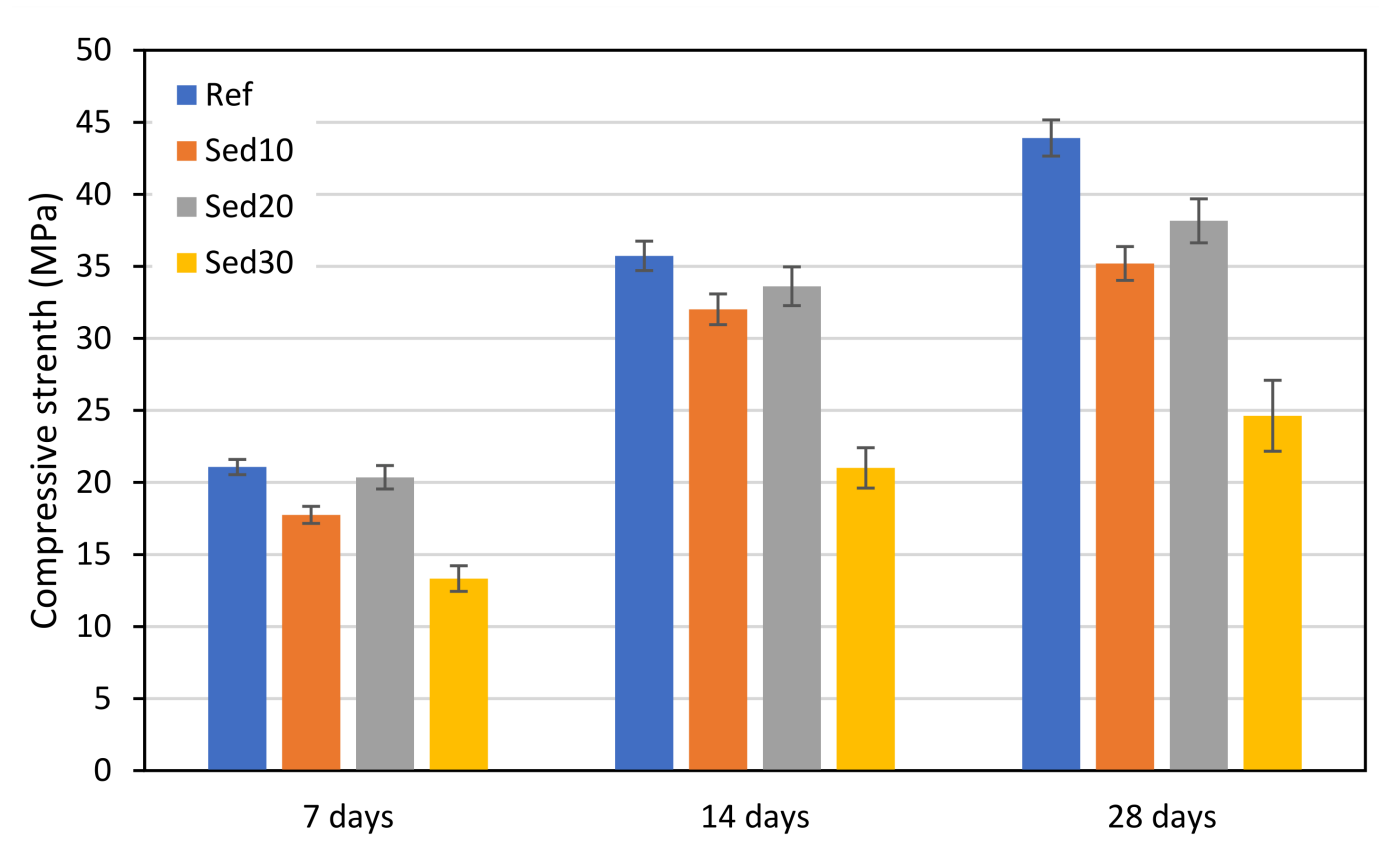
Compressive strength of the different formulations at 7, 14 and 28 days. Error bars represent the standard deviation of the three specimens tested at each age.

The progression of compressive strength values for the formulations under investigation at 7, 14, and 28 days is shown in Figure 4. Strength increased with age, as expected from cement hydration. The reference concrete reached 21.07 MPa, 35.73 MPa, and 43.91 MPa at 7, 14, and 28 days, respectively. The introduction of sediment generally reduced strength, with the magnitude depending on the replacement level. Sed20 reached 20.36 MPa, 33.62 MPa, and 38.16 MPa at 7, 14, and 28 days, respectively. Sed10 reached 17.75 MPa, 32.02 MPa, and 35.20 MPa, while Sed30 reached 13.33 MPa, 21.01 MPa, and 24.63 MPa at 7, 14, and 28 days, respectively.

The findings show that the largest mechanical degradation occurs at the 30% replacement level, while Sed20 retains a comparatively higher strength. Several characteristics of the formulation may contribute to this pattern, including sediment fines, water absorption, organic matter and soluble salts, as well as the simultaneous adjustments in limestone filler and admixture dosage. Increased porosity and water absorption at higher sediment contents are consistent with the observed strength reduction. The comparatively good behavior of Sed20 may reflect a favorable balance of packing and formulation adjustments, but this interpretation remains formulation-specific because the individual contributions of sediment, filler, and admixture were not isolated experimentally.

Contemporary literature predominantly aligns with these trends. Beddaa et al. reported that substituting sand with untreated fine sediments can lead to an average decrease in compressive strength of approximately 50% at a 30% substitution rate [18], which is of the same order of magnitude as the significant degradation observed here for Sed30. Conversely, when the sediment fractions are sandier or partially depleted of silt and organic matter, the impact can be much more limited; Beddaa et al. observed a decrease of approximately 10% for 30% substitutions with selected sandy fractions [27]. Soleimani et al. also show that suitability for valorization depends strongly on the fineness of the sediment, with predominantly sandy sediments retaining the strength class better than fine sediments [2].

### 4.4. Chloride Ion Migration

**Figure 5:**
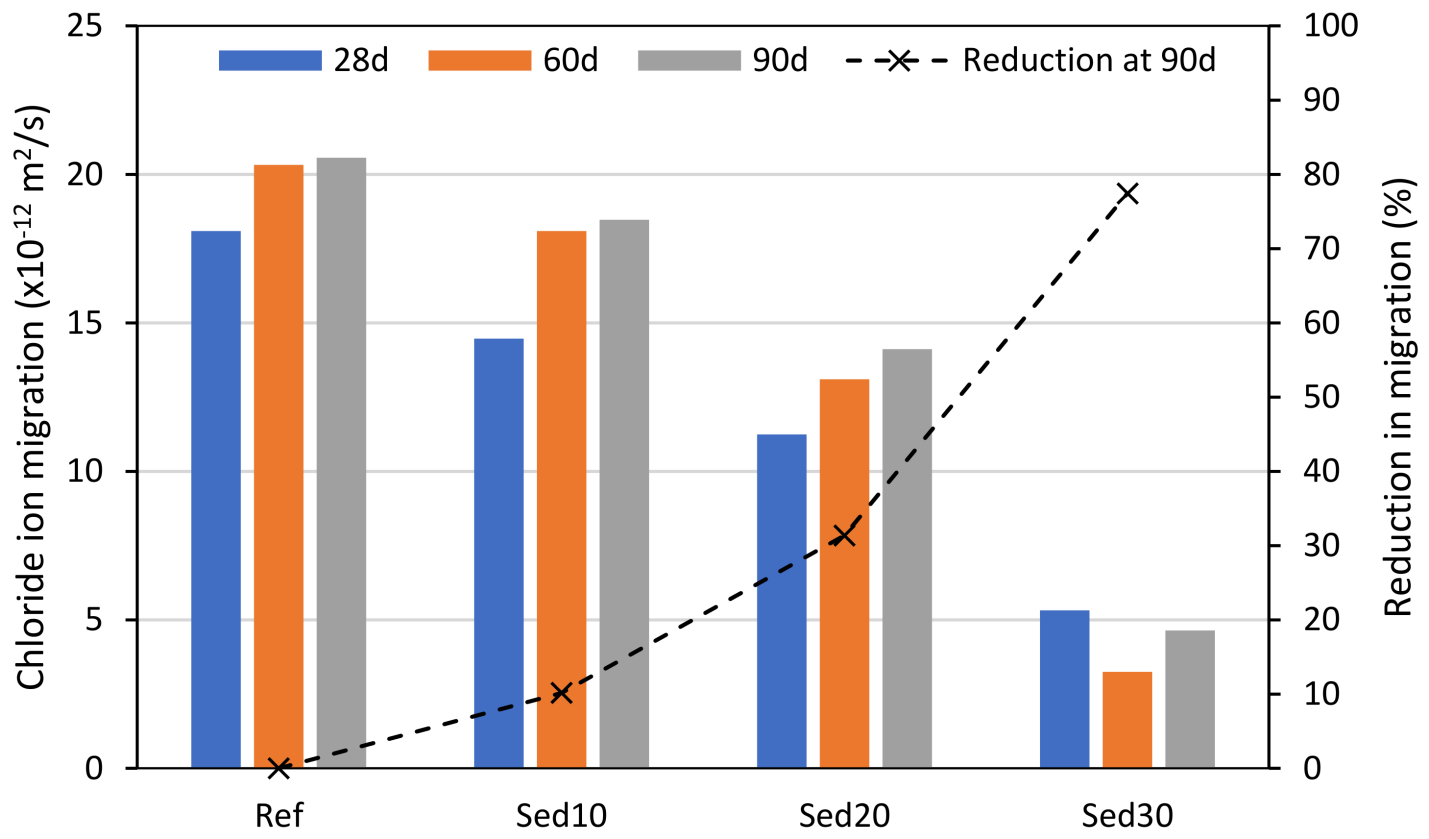
Chloride ion migration coefficient for different formulations.

Figure 5 shows that the apparent chloride ion migration coefficient decreases as the sediment incorporation rate increases. At 90 days, it drops from approximately 20.6 × 10^−12^ m^2^/s for the control to 18.5, 14.1, and 4.6 × 10^−12^ m^2^/s for Sed10, Sed20, and Sed30, respectively. The reduction at 90 days thus reaches approximately 10% for Sed10, 31% for Sed20, and 77% for Sed30 compared to the reference concrete.

The results above indicate a possible improvement in resistance to chloride-ion migration with increasing sediment fractions. This observation may be considered unexpected because, despite increasing water-accessible porosity and wetting absorptiveness, ion migration can depend on pore structure, including tortuosity, and on chloride binding by the matrix [30,31]. In the present study, however, pore-size distribution, connectivity, tortuosity, interfacial-transition-zone morphology, and chloride-binding capacity were not measured. The influence of fines on pore-network tortuosity and chloride binding is therefore presented only as a possible mechanism and requires direct microstructural and chemical verification. In addition, the sediment’s initial chloride content may affect interpretation of the XP P 18-462 migration results; accordingly, these values are used for comparative purposes within the tested series rather than as stand-alone durability compliance indicators.

There have also been similar studies found in the literature on the performance of cement-based materials using sediments. For example, Safhi et al. (2021) have shown that the use of treated sediments improves the microstructural and transport characteristics [28]. Moradi and Shahnoori (2021) have shown that certain durability parameters, including electrical resistivity and waterproofness, are improved when using marine-dredged sediments in concrete, but in relatively small amounts [32]. Similarly, Hayek et al. (2023) suggest that untreated fine marine sediments can be used for marine concrete production if the concrete mixture is properly adjusted [33].

### 4.5. Accelerated Carbonation

**Figure 6:**
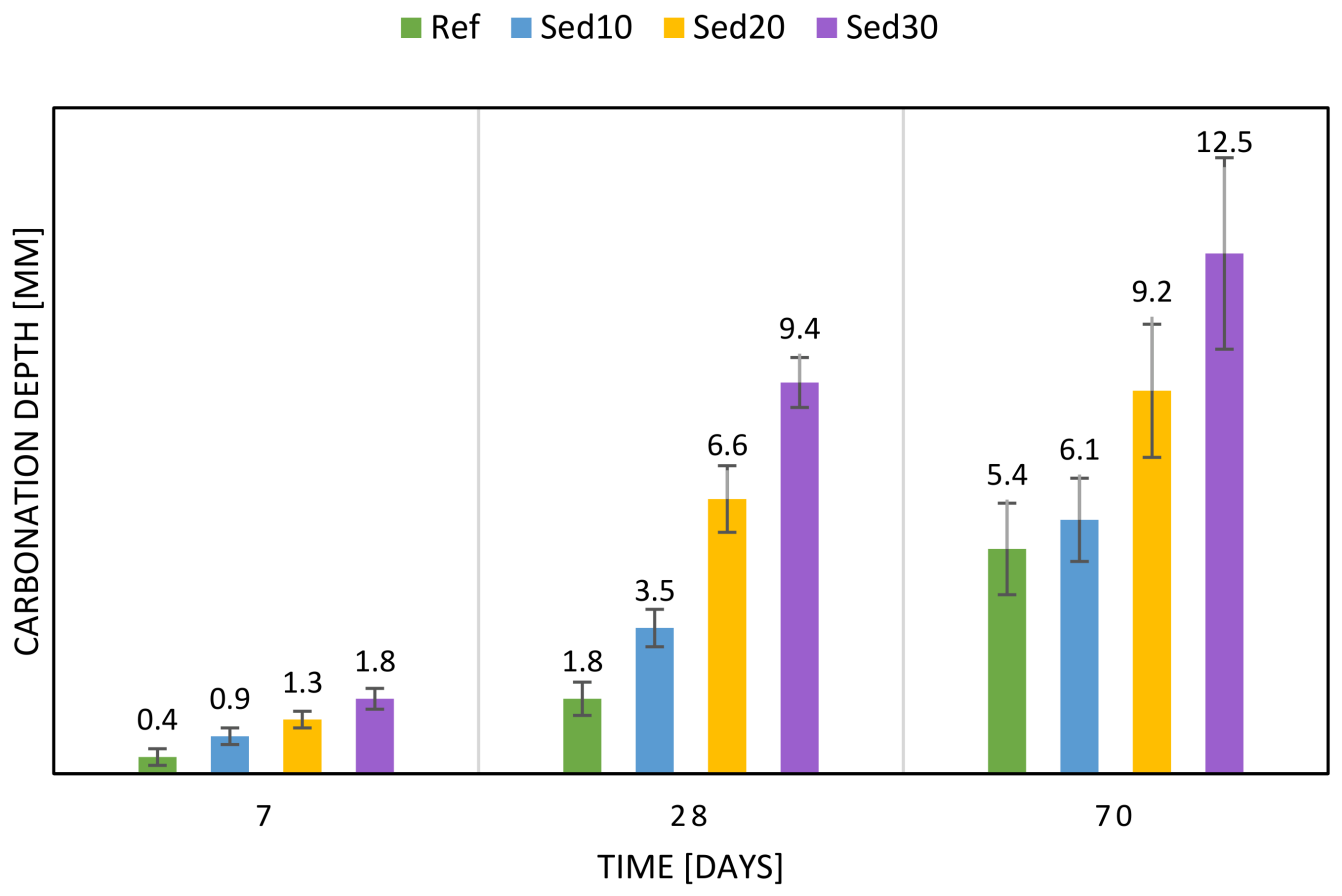
Carbonation depth over time for all formulations. Error bars represent the standard deviation of the replicate depth measurements.

As can be seen from Figure 6, there is an evident trend towards increasing depth of carbonation with time, accompanied by a gradual degradation as the amount of sediment increases. The depth of carbonation at 70 days ranges from 5.4 mm in the control to 12.5 mm in the Sed30 samples. Using the square-root-of-time relationship x = k√t and the mean depths at 7, 28, and 70 days, least-squares fits through the origin give carbonation coefficients k of approximately 0.53, 0.69, 1.10, and 1.52 mm/√day for Ref, Sed10, Sed20, and Sed30, respectively. These coefficients are reported as comparative descriptors of the accelerated test rather than as service-life predictions.

Based on the results shown above, adding raw sediments increases the susceptibility of concrete to carbonation, with a minor impact at a 10% replacement rate, a greater impact at a 20% replacement rate, and an even stronger impact at a 30% replacement rate. This trend corresponds well to other parameters that have been considered in this study, including increased porosity in the form of accessible pores to water, increased immersion absorptivity, increased shrinkage, and decreased compressive strength, which are parameters that promote the entrance of CO_2_ gas into the concrete structure [34]. The high proportion of fine material in the raw sediments, together with their water-absorbency and organic-matter content, can play an important role.

Such findings generally conform to the information reported in previous studies on unprocessed sediments. According to Soleimani et al., partial substitution of the sand component with unprocessed fine sediments can reduce performance even at low concentrations due to the fine nature of the sediment [2]. Similarly, according to Hayek et al., the use of unprocessed fine sediments is permissible only upon proper optimization of the formulation [33]. On the contrary, some positive results can be achieved by using treated sediments in cementitious formulations; for example, according to Ez-zaki et al., adding around 16% treated sediment improves carbonation resistance [35].

### 4.6. Internal Sulfate Swelling

Table 11 shows that internal sulfate swelling increases both over time and with the sediment incorporation rate. Percentage strain values calculated using the full specimen length (L_0_ = 280 mm) are also reported for comparison. At 28 days, swelling increases from 0.38 mm for the reference concrete to 0.49 mm, 0.58 mm, and 0.87 mm for Sed10, Sed20, and Sed30, respectively. At 60 days, it reaches 0.45 mm, 0.57 mm, 0.65 mm, and 0.96 mm, respectively; at 90 days, 0.46 mm, 0.59 mm, 0.69 mm, and 0.98 mm. The increase remains moderate for Sed10 and Sed20 but becomes much more pronounced for Sed30, whose swelling at 90 days is more than twice that of the control.

These results suggest that increasing the incorporation of sediment enhances the concrete’s susceptibility to internal sulfate reactions. Such a development may be related to the presence of soluble sulfates in the raw sediment shown in Table 6, together with increased porosity and water absorption, which can facilitate moisture transport and expansive reactions within the matrix. The literature on internal sulfate attack shows that swelling depends not only on the available sulfate content but also on porosity, alkalinity, and wetting conditions, with ettringite being a principal expansive product associated with deformation [13,36,37]. The trends observed here are therefore consistent with these general mechanisms. However, they should be qualified because the present test is comparative and the available studies mainly concern concretes or mortars containing gypsum-contaminated aggregates or sulfated recycled fine fractions rather than raw dredged sediments.

### 4.7. Leaching of Crushed Specimen

Table 12 highlights a clear effect of the cementitious matrix on the leaching characteristics of crushed concrete, both at 28 days and after environmental exposure. The raw sediment exhibits relatively high concentrations of leachables, including chloride (19,000 mg/kg), sulfate (4000 mg/kg), and fluoride (13 mg/kg). After incorporation into concrete and subsequent crushing at 28 days, the measured levels drop sharply: chlorides to 31–828 mg/kg, sulfates to 40–76.1 mg/kg, and fluorides to 5–6.2 mg/kg, while most trace metals remain at very low levels. This decrease is consistent with stabilization/solidification mechanisms widely described for cementitious matrices, including physical encapsulation, adsorption, ion exchange, and incorporation into cement hydrates [1,16,38]. After environmental monitoring, leaching of crushed material shows that this stabilization remains partial: chlorides decrease further (6–437 mg/kg), while fluorides increase in sediment-containing formulations, from 25 mg/kg for Sed10 to 54 mg/kg for Sed30, and nickel reaches 0.4 mg/kg for Sed20 and 0.6 mg/kg for Sed30.

From a regulatory-screening standpoint, all crushed concrete formulations remain well below the non-hazardous waste thresholds, but several exceedances of the inert threshold persist, notably for chlorides in Sed30 at 28 days (828 mg/kg > 800 mg/kg), for fluorides after environmental monitoring in concretes containing sediment, and for nickel in Sed30. Sed20 reaches the nickel inert-waste threshold (0.4 mg/kg) but does not exceed it. This behavior is consistent with studies showing that leaching from cementitious materials is highly dependent on pH, curing time, monolithic or crushed state, and prior exposure conditions [16,39,40]. The results therefore show that incorporation into concrete reduces the mobility of several pollutants present in the raw sediment but does not eliminate end-of-life release risks, particularly for the most heavily loaded formulation. Because only the EN 12457-2 test condition was evaluated, pH-dependent release boundaries and long-term cumulative leaching cannot be established from the present data.

### 4.8. Environmental Monitoring Under Simulated Rain

Table 13 presents the concentrations measured in runoff water collected after 1, 3, 6, 13, 27, 41, 55, 83, 111, and 139 days of exposure under simulated rainfall. In this preliminary single-slab assessment, releases remained low for all formulations and were below the adopted screening thresholds at all reported time intervals. The predominant species released are chlorides and sulfates, with concentrations ranging from 36 to 83 mg/L and 39.1 to 126 mg/L for the reference concrete, and with similar ranges for Sed10, Sed20, and Sed30, without a systematic increase with sediment content. Fluoride also remains low, between 0.20 and 0.42 mg/L, below the adopted limit values. For trace elements, most concentrations are very low or below the limits of quantification. Because runoff concentrations were measured only at the discrete sampling dates reported in Table 13 rather than for every daily runoff batch, cumulative mass release per unit surface area cannot be reconstructed reliably from the retained dataset. The environmental results are therefore interpreted as preliminary concentration-based comparative indicators.

These preliminary results show that, under the simulated rainfall conditions investigated, the inclusion of raw sediments does not show a systematic deterioration of runoff water quality compared with the reference concrete. This is consistent with partial immobilization of the elements of interest by the cementitious matrix through encapsulation and physicochemical interactions [1,16]. The observation is also consistent with studies on monolithic cementitious materials showing relatively low releases under controlled exposure conditions, in contrast with crushed specimens whose mobility can increase after loss of matrix integrity [40,41,42]. Fluctuations during the test, especially in chlorides and sulfates from day 55 to day 83, may reflect progressive surface leaching rather than deterioration of the material itself.

## 5. Study Limitations and Scope

The formulation strategy intentionally adjusted limestone filler and superplasticizer dosages to maintain the target consistency class as sediment replacement increased. This allows comparison of workable sediment-containing concretes, but it introduces co-varying formulation variables and prevents strict attribution of every performance change to sediment alone. A full economic assessment including sediment conditioning, admixture demand, performance-related service-life effects, and life-cycle cost was not available; consequently, no cost-optimal dosage is claimed.

The mechanisms proposed for chloride migration are hypotheses because pore-size distribution, pore connectivity/tortuosity, interfacial-transition-zone morphology, and chloride-binding capacity were not measured directly. Likewise, the end-of-life assessment is limited to EN 12457-2 conditions, and the single-pass rainfall dataset with discrete chemical sampling dates was not designed for a complete cumulative mass balance. These limitations do not alter the comparative experimental observations but define the boundary within which the conclusions should be interpreted. Individual slump-flow values were not retained, so the SF2 classification cannot be independently verified. In addition, the simulated-rainfall test used one slab per formulation; the corresponding environmental observations are preliminary and should not be generalized beyond the conditions investigated.

## 6. Conclusions

The purpose of this case study was to evaluate the feasibility of using raw marine sediments from port zones as a sand substitute in concrete. An experimental technique was used that accounted for mechanical and physical properties, durability, the impact of artificial rain, and post-mortem assessment of crushed concrete.

The effectiveness of the tested marine sediments strongly depends on their amount. At a 20% replacement level, the concrete retained comparatively acceptable measured properties within the tested series: its compressive strength after 28 days was 38.16 MPa, compared with 43.91 MPa for the control; water-accessible porosity was 12.66%, and water absorption was 5.62%. At 30%, the effect was much stronger: strength dropped to 24.63 MPa, porosity rose to 14.27%, and absorption rose to 7.41%. Shrinkage reached 783 µm/m at 90 days, compared with 311 µm/m for the control, and carbonation depth at 70 days reached 12.5 mm, compared with 5.4 mm for the reference concrete. The present work did not test the crushed concrete as recycled aggregate; therefore, these results should not be interpreted as direct evidence of recyclability.

Taking into account the environmental results, the tests demonstrate a decrease in the leachability of several species compared with the untreated sediment, especially for chlorides (19,000 mg/kg in raw sediment against 324 mg/kg for Sed20 and 828 mg/kg for Sed30 after 28 days) and sulfates (4000 mg/kg in raw sediment against 50 mg/kg for Sed20). In the preliminary simulated-rainfall assessment, which used one slab per formulation, runoff concentrations remained within the adopted screening criteria and showed no systematic deterioration compared with the control sample. The leaching of crushed concrete nevertheless shows that stabilization is incomplete, with remaining concerns for fluorides and, at the highest replacement level, nickel.

Consequently, in this particular case study, 20% sediment provided the best overall balance among the sediment-containing mixtures investigated in terms of resource substitution, measured technical performance, and the preliminary environmental observations. This statement is not the result of a formal optimization or life-cycle cost analysis. Increasing the replacement level to 30% produced substantial losses in mechanical and durability-related performance and greater end-of-life release concerns. These conclusions should be interpreted as specific to the sediment studied, the adjusted cementitious formulations, and the experimental conditions applied, and not as a generalization to all dredged sediments or engineering applications.

## Figures and Tables

**Table 1:** Chemical, physical, and mechanical properties of CEM II/A-LL 52.5 R cement.

Category	Parameter	Value
**Chemical characteristics [%]**	SO^3^	2.5
	MgO	0.7
	Na_2_O	0.2
	Cl^−^	0.02
	Fire loss	1.1
**Physical properties**	Blaine surface area [cm^2^/g]	3850
	Absolute Density [kg/m^3^]	3030
**Compressive strength [MPa]**	1d	21–28
	2d	34–42
	28d	56–66

**Table 2:** Physicochemical and particle size characteristics of the limestone filler.

Category	Parameter	Value
**Component**	CaCO_3_	98.2%
	Cl^−^	0.003%
	Sulfates	0.01%
	Total silica	0.02
	Others	≈1.77%
**Particle size**	Particles < 0.125 mm	98%
	Particles < 0.063 mm	90%
**Density**	Density	2700 kg/m^3^

**Table 3:** Physical properties of the 0/4 washed sand used as a reference fine aggregate.

Parameter	Value
**Density**	2630 kg/m^3^
**Water absorption**	0.1%
**Sand equivalent**	81.3
**Fineness modulus**	2.3

**Table 4:** Physical properties of the washed semi-crushed gravel used as a coarse aggregate.

Parameter	Value
**Density**	2460 kg/m^3^
**Water absorption**	2.4%
**Freeze/thaw resistance**	0.2
**Los Angeles**	16.0

**Table 5:** Granulometric, physical and geotechnical characteristics of the raw marine sediment studied.

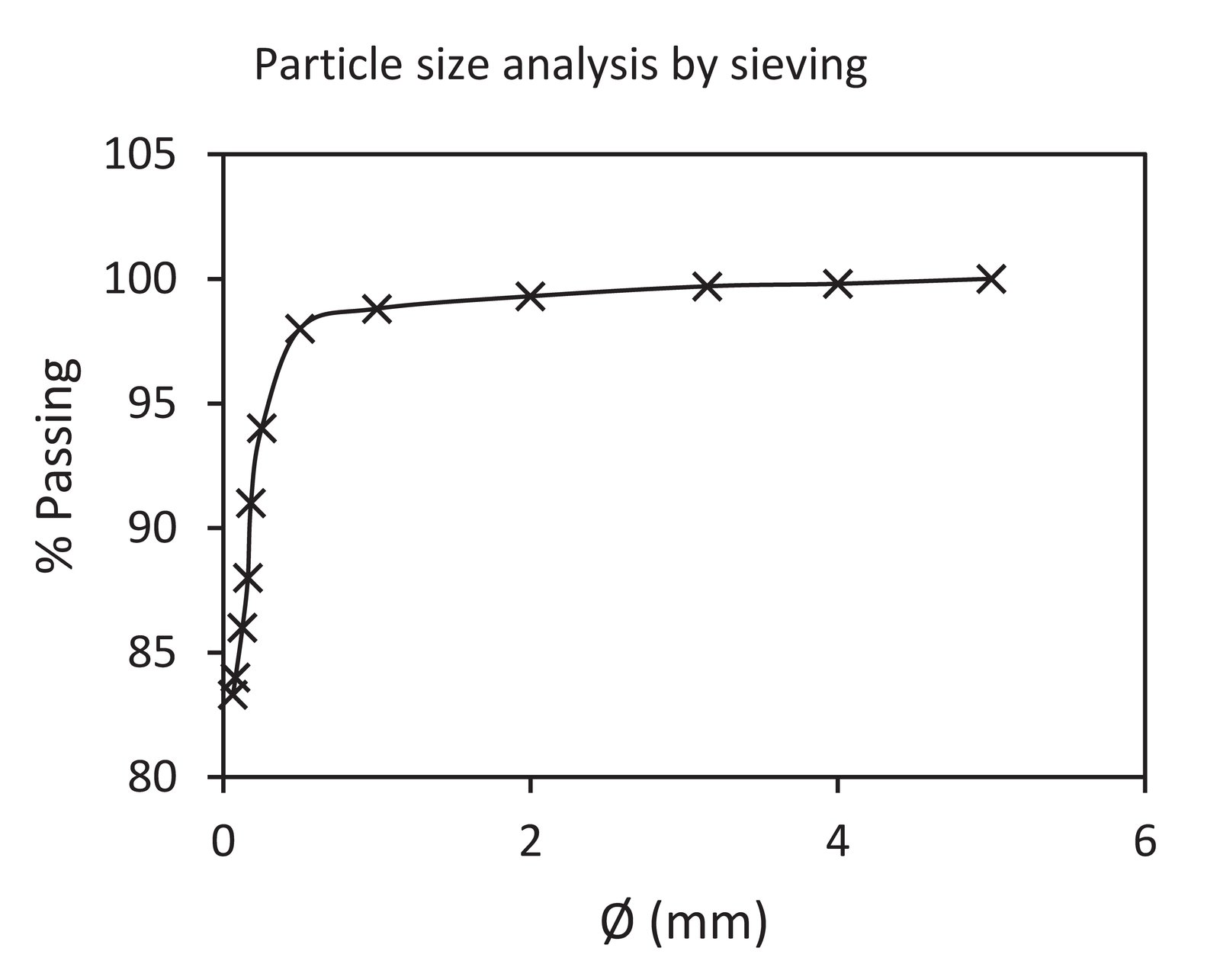		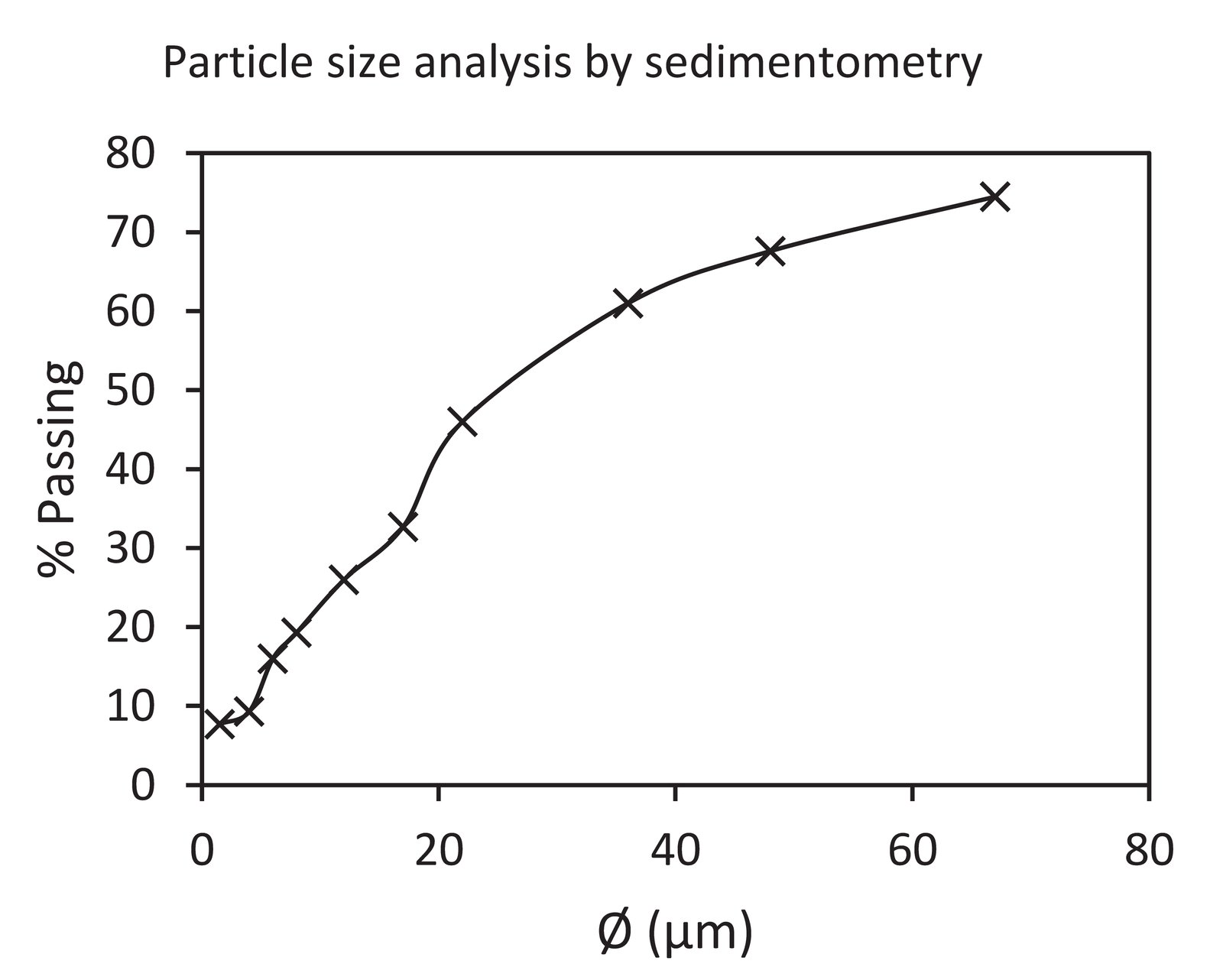	
Category	Parameter	Unit	Sediment
General	Loss on ignition at 550 °C	% Dry mass	10.64
	Value in methylene blue	g per 100 g of blue	1.31
	Permeability coefficient	m/s	5 × 10^−9^
Atterberg limit	Consistency index	-	−0.88
	Liquidity limit	-	62
	Plasticity index	-	20
General	Water content	%	22.1
	Actual pre-dried density	kg/m^3^	2680
	Dry surface saturated density	kg/m^3^	2950
	True density (oven-dried)	kg/m^3^	2830
	Absolute density	kg/m^3^	3220
	Water absorption	%	4.3
	Effect on cement setting time	min	140
	Sand friability coefficient	-	41
	Fine particle content	%	84.9

**Table 6:** Chemical composition and metal content of the raw marine sediment studied.

Category	Parameter	Sediment
General	Total sulfur	0.33% S
	Water-soluble sulfates	0.34% SO_4_
	Acid-soluble sulfates	0.31% SO_3_
	Water-soluble chlorides	1.508%
	Humic matter content	8.84%
Oxide composition of the ash	Silicon dioxide SiO_2_	56.59%
	Iron Fe_2_O_3_	2.65%
	Aluminum Al_2_O_3_	2.33%
	Calcium oxide CaO	2.73%
	Magnesium MgO	0.92%
	Sodium Na_2_O	1.44%
	Potassium K_2_O	0.31%
	Titanium dioxide TiO_2_	0.031%
	Manganese MnO_2_	0.017%
	Phosphorus P_2_O_5_	0.12%
	Sulfur SO_3_	0.67%
	Total nitrogen N	0.4%
Metal content	Arsenic As	9 mg/kg dry matter
	Cadmium Cd	<2.00 mg/kg dry matter
	Copper Cu	72 mg/kg dry matter
	Mercury Hg	<0.50 mg/kg dry matter
	Molybdenum Mo	5 mg/kg dry matter
	Nickel Ni	17 mg/kg dry matter
	Lead Pb	40 mg/kg dry matter
	Antimony Sb	<5.00 mg/kg dry matter
	Zinc Zn	172 mg/kg dry matter
	Chrome Cr	83 mg/kg dry matter

**Table 7:** Environmental quality indicators and leaching results of the raw marine sediment studied. Colored cells indicate the regulatory class for which the measured sediment value satisfies the corresponding threshold.

Parameter	Threshold			Sediment
	ISDI Class 3	ISDND Class 2	ISDD Class 1	
**General physicochemical properties**				
Dry matter	>30	>30	> 30	51%wt
TOC	<30,000	<50,000	-	44,000 mg/kg dry matter
pH (KCl)	-	-	-	8.2
Temperature for MES pH	-	-	-	19.2 °C
**Organic contaminants**				
Total BTEX	<6	<30	-	<0.25 mg/kg dry matter
Sum of PAHs (10)—VROM	<50	<100	-	3.3 mg/kg dry matter
Total PAHs (16)—EPA	<50	<100	-	4.7 mg/kg dry matter
Total PCBs (7)	<1	<50	-	0.018 mg/kg dry matter
C10–C40 hydrocarbons	<500	<5000	-	250 mg/kg dry matter
**Leaching test conditions and eluate properties**				
L/S	-	-	-	10 mL/g
Final pH after leaching	-	-	Between 4 and 13	8.1
Temperature for MES pH	-	-	-	18.1 °C
Conductivity (25 °C) after leaching	-	-	-	6 710 µS/cm
**Organic indicators in the eluate**				
COD, TOC in eluate	<500	<800	<1000	150 mg/kg dry matter
**Metals and metalloids in the eluate**				
Antimony	<0.06	<0.7		0.048 mg/kg dry matter
Arsenic	<0.5	<2	<25	0.03 mg/kg dry matter
Barium	<20	<100	<300	0.25 mg/kg dry matter
Cadmium	<0.04	<1	<5	<0.002 mg/kg dry matter
Chromium	<0.5	<10	<70	<0.01 mg/kg dry matter
Copper	<2	<50	<100	<0.02 mg/kg dry matter
Mercury	<0.01	<0.2	<2	<0.0005 mg/kg dry matter
Lead	<0.5	<10	<50	<0.02 mg/kg dry matter
Molybdenum	<0.5	<10	<30	1 mg/kg dry matter
Nickel	<0.4	<10	<40	<0.03 mg/kg dry matter
Selenium	<0.1	<0.5	<7	<0.02 mg/kg dry matter
Zinc	<4	<50	<200	<0.1 mg/kg dry matter
**Other eluate parameters**				
Fraction soluble	<4000	<60,000	-	38,500 mg/kg dry matter
Phenol index	<1	<10	-	<0.1 mg/kg dry matter
Fluorides	<10	<150	<500	13 mg/kg dry matter
Chlorides	<800	<15,000	-	19,000 mg/kg dry matter
Sulfates	<1000	<20,000	-	4000 mg/kg dry matter

**Table 8:** Mix compositions (kg/m^3^).

Component (kg/m^3^)	Ref	Sed10	Sed20	Sed30
**Sand**	885	796	708	620
**Dredged sediment**	0	89	177	265
**Gravel**	820	820	820	820
**Cement**	330	330	330	330
**Limestone Filler**	130	140	150	160
**Admixture**	2	3	7	9
**Effective water**	150	150	150	150

**Table 9:** Data from the environmental monitoring protocol under simulated rainfall.

Fixed Input Data	Value	Unit
**1991–2020 mean annual precipitation aggregated over France**	935	L/m^2^/year
**Volume of rainwater applied per day**	12	L/0.25 m^2^/day
**Follow-up time**	139 days	

**Table 10:** Main physical properties of concretes formulated with partial substitution of sand by raw sediments.

Formulation	Ref	Sed10	Sed20	Sed30
**Porosity [%]**	11.46	11.92	12.66	14.27
**Density [kg/m^3^]**	2443	2431	2419	2376
**Water absorption by immersion [%]**	4.33	5.13	5.62	7.41

**Table 11:** Internal sulfate swelling for different formulations.

Internal Sulfate Swelling ΔL (mm)/Strain (%)	Ref	Sed10	Sed20	Sed30
**28d**	0.38 0.136%	0.49 0.175%	0.58 0.207%	0.87 0.311%
**60d**	0.45 0.161%	0.57 0.204%	0.65 0.232%	0.96 0.343%
**90d**	0.46 0.164%	0.59 0.211%	0.69 0.246%	0.98 0.350%

**Table 12:** Leaching behavior of crushed concrete, both at 28 days and after environmental monitoring (Values are highlighted when it pass the limit value by the Class color).

Parameters	Leaching of Raw Sediment	Leaching of Crushed Material at 28 Days (mg/kg)				Leaching of Crushed Material After Environmental Monitoring (mg/kg)				Limit Value (mg/kg)		
										ISDI *	ISDND *	ISDD *
		Ref	Sed10	Sed20	Sed30	Ref	Sed10	Sed20	Sed30	Inert Waste Storage	Non-Hazardous Waste Storage Facility	Hazardous Waste Storage Facility
Chlorides	19,000	31	198	324	828	6	114	198	437	800	15,000	25,000
Sulfates	4000	40	50	50	76.1	40	46	50	64	1000	1000	20,000
Fluorides	13	5	5.6	6	6.2	5	25	42	54	10	150	500
Arsenic (As)	0.03	<0.1	<0.1	<0.1	0.0124	<0.1	<0.1	<0.1	<0.1	0.5	2	25
Barium (Ba)	0.25	0.3	1.1	1.13	0.957	0.5	0.9	1.09	0.83	20	100	300
Cadmium (Cd)	<0.002	<0.002	<0.002	<0.002	<0.002	<0.002	<0.009	<0.009	<0.009	0.04	0.5	1
Chrome (Cr)	<0.01	0.001	0.0091	0.0163	0.0194	0.001	0.01	0.02	0.02	0.5	2	10
Copper (Cu)	<0.02	0.03	0.152	0.222	0.543	0.03	0.1	0.12	0.22	2	2	50
Molybdenum (Mo)	1	<0.09	<0.09	<0.09	0.0959	<0.09	<0.09	<0.09	<0.09	0.5	2	10
Nickel (Ni)	<0.03	<0.02	<0.02	0.426	0.593	0.05	0.2	0.4	0.6	0.4	2	10
Lead (Pb)	<0.02	<0.03	<0.03	<0.03	<0.03	<0.03	<0.03	<0.03	<0.03	0.5	2	50
Antimony (Sb)	0.048	<0.002	<0.002	<0.002	<0.002	<0.002	<0.06	<0.06	<0.06	0.06	0.3	5
Selenium (Se)	<0.02	<0.005	<0.005	<0.005	0.0057	<0.005	<0.005	<0.08	<0.08	0.1	0.5	7
Zinc (Zn)	<0.05	<0.05	<0.05	<0.05	<0.05	<0.01	<0.01	<0.01	<0.01	4	4	100

* Abbreviation of French origin in accordance with regulations: ISDI: Installation de Stockage de Déchets Inertes; ISDND: Installation de Stockage de Déchets Non Dangereux; ISDD: Installation de Stockage de Déchets Dangereux.

**Table 13:** Anion and trace-element concentrations measured in runoff water collected after 1, 3, 6, 13, 27, 41, 55, 83, 111 and 139 days of exposure under simulated rain. * Environmental Quality Standards (EQS), including the French Order of 11 January 2007 on surface water quality limits for drinking-water production, pursuant to Articles R.1321-38 to R.1321-41.

**Ref**	**Collecting Interval (Day)**	**1**	**3**	**6**	**13**	**27**	**41**	**55**	**83**	**111**	**139**	* Limit
	Chlorides (mg/L)	49	41.2	36	46	58	55	49	83	77	65	200
	Sulfates (mg/L)	78.3	48	39.1	80	102	95	125	126	125	105	150
	Fluorides (mg/L)	0.25	0.23	0.32	0.3	0.4	0.4	0.42	0.42	0.38	0.38	0.7–1.7
	Arsenic (µg/L)	<5	<5	<5	<11	<11	<11	<11	<11	<11	<11	50
	Cadmium (µg/L)	<5	<5	<5	<0.9	<0.9	<0.9	<0.9	<0.9	<0.9	<0.9	5
	Chromium (µg/L)	<5	<5	<5	<0.4	<0.4	<0.4	<0.4	<0.4	<0.4	<0.4	50
	Copper (µg/L)	140	200	100	6.3	12	13	17	17	15	12	1000
	Nickel (µg/L)	6	<5	<5	<4.7	<4.7	<4.7	12	12	11	11	400
	Lead (µg/L)	<5	<5	<5	<3.2	<3.2	<3.2	<3.2	<3.2	<3.2	<3.2	50
	Zinc (µg/L)	80	20	20	27	14	8.1	26	30	23	20	5000
	Antimony (µg/L)	0.23	0.24	<0.20	<5.7	<5.7	<5.7	<5.7	<5.7	<5.7	<5.7	60
	Barium (µg/L)	32.6	23.7	20.3	20	24	23	44	44	44	44	1000
	Molybdenum (µg/L)	0.82	0.79	0.31	<8.8	<8.8	<8.8	<8.8	<8.8	<8.8	<8.8	500
	Selenium (µg/L)	2.76	1.81	0.58	<8.3	<8.3	<8.3	12	9.9	<8.3	<8.3	100
**Sed10**	**Collecting Interval (Day)**	**1**	**3**	**6**	**13**	**27**	**41**	**55**	**83**	**111**	**139**	* Limit
	Chlorides (mg/L)	46.5	39.7	37.1	51.5	51.5	46.5	55	76.5	75	57.5	200
	Sulfates (mg/L)	73.8	44.8	40.4	88	84	72.5	123	124	122.5	88	150
	Fluorides (mg/L)	0.24	0.3	0.35	0.3	0.35	0.35	0.42	0.42	0.4	0.4	0.7–1.7
	Arsenic (µg/L)	<5	<5	<5	<11	<11	<11	<11	<11	<11	<11	50
	Cadmium (µg/L)	<5	<5	<5	<0.9	<0.9	<0.9	<0.9	<0.9	<0.9	<0.9	5
	Chromium (µg/L)	<5	<5	<5	<0.4	<0.4	<0.4	<0.4	<0.4	<0.4	<0.4	50
	Copper (µg/L)	70	50	40	45	3	<2.1	20	20	19	18	1000
	Nickel (µg/L)	<5	<5	<5	<4.7	<4.7	<4.7	12	12	12	12	400
	Lead (µg/L)	<5	<5	<5	<3.2	<3.2	<3.2	<3.2	<3.2	<3.2	<3.2	50
	Zinc (µg/L)	35	35	37	30	35	36	42	43	45	40	5000
	Antimony (µg/L)	<0.20	<0.20	<0.20	<5.7	<5.7	<5.7	<5.7	<5.7	<5.7	<5.7	60
	Barium (µg/L)	15	17	15	20	19	19	32	32	32	31	1000
	Molybdenum (µg/L)	0.4	0.23	0.2	<8.8	<8.8	<8.8	<8.8	<8.8	<8.8	<8.8	500
	Selenium (µg/L)	<0.50	0.8	0.8	<8.3	<8.3	<8.3	<8.3	<8.3	<8.3	<8.3	100
**Sed20**	**Collecting Interval (Day)**	**1**	**3**	**6**	**13**	**27**	**41**	**55**	**83**	**111**	**139**	* Limit
	Chlorides (mg/L)	43.9	38.1	38.1	57	45	38	61	70	73	50	200
	Sulfates (mg/L)	69.3	41.6	41.6	96	66	50	121	122	120	71	150
	Fluorides (mg/L)	0.22	0.37	0.37	0.3	0.3	0.3	0.42	0.41	0.41	0.41	0.7–1.7
	Arsenic (µg/L)	<5	<5	<5	<11	<11	<11	<11	<11	<11	<11	50
	Cadmium (µg/L)	<5	<5	<5	<0.9	<0.9	<0.9	<0.9	<0.9	<0.9	<0.9	5
	Chromium (µg/L)	<5	<5	<5	<0.4	<0.4	<0.4	<0.4	<0.4	<0.4	<0.4	50
	Copper (µg/L)	70	20	20	28	3	<2.1	31	15	17	12	1000
	Nickel (µg/L)	<5	<5	<5	<4.7	<4.7	<4.7	13	13	12	12	400
	Lead (µg/L)	<5	<5	<5	<3.2	<3.2	<3.2	<3.2	<3.2	<3.2	<3.2	50
	Zinc (µg/L)	40	20	20	59	<1.0	<5.7	47	21	26	20	5000
	Antimony (µg/L)	0.2	<0.20	<0.20	<5.7	<5.7	<5.7	<5.7	<5.7	<5.7	<5.7	60
	Barium (µg/L)	27.8	15.5	15.5	25	18	16	43	44	43	32	1000
	Molybdenum (µg/L)	0.46	0.3	0.3	<8.8	<8.8	<8.8	<8.8	<8.8	<8.8	<8.8	500
	Selenium (µg/L)	2.03	<0.50	<0.50	<8.3	<8.3	<8.3	<8.3	<8.8	<8.3	<8.3	100
**Sed30**	**Collecting Interval (Day)**	**1**	**3**	**6**	**13**	**27**	**41**	**55**	**83**	**111**	**139**	* Limit
	Chlorides (mg/L)	44.6	42.9	38.5	48	38	38	66	49	49	37	200
	Sulfates (mg/L)	53.2	44.7	43.2	86	56	52	122	123	123	62	150
	Fluorides (mg/L)	0.2	0.21	0.23	0.3	0.3	0.3	0.4	0.4	0.4	0.4	0.7–1.7
	Arsenic (µg/L)	<5	<5	<5	<11	<11	<11	<11	<11	<11	<11	50
	Cadmium (µg/L)	<5	<5	<5	<0.9	<0.9	<0.9	<0.9	<0.9	<0.9	<0.9	5
	Chromium (µg/L)	<5	<5	<5	<0.4	<0.4	<0.4	<0.4	<0.4	<0.4	<0.4	50
	Copper (µg/L)	70	70	40	43	2.4	<2.1	15	18	19	18	1000
	Nickel (µg/L)	7	<5	<5	<4.7	<4.7	<4.7	12	12	13	13	400
	Lead (µg/L)	<5	<5	<5	<3.2	<3.2	<3.2	<3.2	<3.2	<3.2	<3.2	50
	Zinc (µg/L)	50	50	30	33	<1	<1	22	<5.7	23	14	5000
	Antimony (µg/L)	0.25	0.22	<0.20	<5.7	<5.7	<5.7	<5.7	21	<5.7	<5.7	60
	Barium (µg/L)	25.3	14.6	18.2	19	16	16	43	<8.8	44	37	1000
	Molybdenum (µg/L)	0.41	0.48	0.29	<8.8	<8.8	<8.8	<8.8	<8.8	<8.8	<8.8	500
	Selenium (µg/L)	1.91	1.33	0.8	<8.3	<8.3	<8.3	9.2	<8.8	<8.3	<8.3	100

## Data Availability

The original contributions presented in this study are included in the article. Further inquiries can be directed to the corresponding author.
